# Resting state brain dynamics and its transients: a combined TMS-EEG study

**DOI:** 10.1038/srep31220

**Published:** 2016-08-04

**Authors:** Mireille Bonnard, Sophie Chen, Jérôme Gaychet, Marcel Carrere, Marmaduke Woodman, Bernard Giusiano, Viktor Jirsa

**Affiliations:** 1 Aix Marseille Université, INS, Institut de Neurosciences des systèmes, Marseille, France. Inserm,UMR_S 1106, 13005, Marseille, France

## Abstract

The brain at rest exhibits a spatio-temporally rich dynamics which adheres to systematic behaviours that persist in task paradigms but appear altered in disease. Despite this hypothesis, many rest state paradigms do not act directly upon the rest state and therefore cannot confirm hypotheses about its mechanisms. To address this challenge, we combined transcranial magnetic stimulation (TMS) and electroencephalography (EEG) to study brain’s relaxation toward rest following a transient perturbation. Specifically, TMS targeted either the medial prefrontal cortex (MPFC), i.e. part of the Default Mode Network (DMN) or the superior parietal lobule (SPL), involved in the Dorsal Attention Network. TMS was triggered by a given brain state, namely an increase in occipital alpha rhythm power. Following the initial TMS-Evoked Potential, TMS at MPFC enhances the induced occipital alpha rhythm, called Event Related Synchronisation, with a longer transient lifetime than TMS at SPL, and a higher amplitude. Our findings show a strong coupling between MPFC and the occipital alpha power. Although the rest state is organized around a core of resting state networks, the DMN functionally takes a special role among these resting state networks.

The existence of Resting State Networks (RSNs) is now clearly established^1^,^2^,^3^,^4^. When human beings stay motionless without falling asleep and let their thoughts run freely without engaging in any task, such as attention^5^,^6^, perception^7^,^8^ or action^9^, a consistent network organization, i.e. well-organized spatiotemporal pattern, is observed in brain activation signals^10^,^11^,^12^,^13^,^14^. Several studies applied group-level independent component analysis (ICA), finding 6 to 10 RSNs, each consisting of a set of brain regions exhibiting highly coherent activity within the network. This organization has been reported in several imaging modalities: blood oxygen level dependent (BOLD) signals^10^,^11^,^12^,^14^, simultaneous functional Magnetic Resonance Imaging (fMRI) and electroencephalography (EEG)^2^,^15^, magnetoencephalography (MEG)^3^,^16^, stereotactic electroencephalography (SEEG)^17^,^18^, and Transcranial Magnetic Stimulation (TMS) combined to EEG^19^,^20^,^21^. While most RSNs correlate with one or more task conditions (“task positive”), the so-called default mode network (DMN) correlates negatively with task conditions and activates during rest^12^. The DMN comprises bilaterally the medial prefrontal cortex, the inferior parietal lobule -mainly the angular gyrus- and the posterior cingulate cortex^1^,^2^.

While the relation among electrophysiological rhythms and RSNs activations is unclear, available evidence suggests that alpha power is positively correlated to the DMN^22^,^23^,^24^. Initial rest-state studies using simultaneous EEG/fMRI attempted to relate changes in EEG power in several frequency bands to changes in the BOLD signal^2^,^15^. Alpha oscillations were found to correlate negatively with either the occipital and parietal area^22^,^23^ or with frontal and parietal area^15^,^24^ depending on subjects and/or sessions^25^. Mantini and collaborators^2^ showed that several rhythms correlate with each functional RSN: the BOLD signal in the DMN positively correlated to both alpha and beta rhythms, while the Dorsal Attentional Network (DAN) showed negative correlations with alpha and beta rhythms. Moreover, during the resting state, when posterior alpha power increases, BOLD decreases in the primary visual cortex and increases in the medial prefrontal, posterior cingulate cortex and left lateral inferior cortex, i.e. all parts of the DMN^26^, as well as the thalamus^23^,^27^,^28^. Finally, ICA applied to MEG data^3^,^16^ also showed alpha power positively correlated with DMN activity.

The hypothesis that alpha power is linked to the rest state is corroborated by “activation” paradigms showing an inverse relation between pre-stimulus alpha power over occipital sites and the performance in perceptual visual tasks^8^, and visual cortex excitability^7^. The DMN presence has been confirmed in SEEG where broadband gamma power in two well-identified nodes of the DMN (medial prefrontal cortex and posterior cingulate cortex) increased during resting with simple fixation and decreases when subjects engage movement, or target detection^18^, with an amplitude of decrease depending on task difficulty and having some influence on the performance level^29^. The same decrease was observed for the alpha rhythm^29^. This leads to the hypothesis that pre-stimulus alpha oscillation modulates many cognitive functions^30^,^31^: the lowest the alpha, the highest the performance. These findings from both the rest state and activation paradigm literature suggest that posterior alpha power correlates positively with DMN activity and negatively with task performance. However, to date, this correlation has not been examined rigorously under an experimental paradigm able to establish a tight relation between alpha power and rest state.

As stated by Deco and collaborators^32^, “RSNs dynamics is the gateway to the array of cognitive architectures that the brain has available”. In this theoretical perspective, RSNs represent exploration by the brain of what is possible, the so-called brain’s dynamic repertoire^33^. Regarding the underlying mechanisms generating the oscillations at rest^34^, most of the proposals claim that the large scale brain network dynamics operates close to instability, that is to say the resting state dynamics is critical. Implications of this claim are in particular remarkable regarding the meaning of the resting state in reference to information processing: criticality means that the brain operates close to a threshold at which new functionally relevant brain states can be easily created^32^. If the resting state dynamics of the brain is indeed critical, then there will exist certain sub-networks that express themselves stronger and on a slower time scale than other sub-networks. In this perspective, exogenous drives, such as TMS, offer independent means of testing understanding of RSNs dynamics based on perturbation^32^.

To test the hypotheses that alpha power is linked to the DMN, we used alpha-power triggered TMS to perturb^35^,^36^ nodes of different RSNs. Specifically, we applied single pulse stereotactic TMS to either the medial prefrontal cortex (MPFC), a node of the DMN^3^,^12^, or the superior parietal lobule (SPL), a node of task positive networks, often cited as a part of the DAN^5^,^6^. We analyzed the result in terms of TMS-Evoked Potential (TEP, as an index of system’s perturbation) and induced alpha response (Event Related Desynchronisation^37^, ERD, or Event Related Synchronisation, ERS) allowing the system to restore its initial dynamics. A key innovation of the present study is the use of spontaneous increases in occipital alpha power, analyzed online, in order to trigger TMS. Based on the hypothesis that alpha power and DMN are tightly related, we predict that the alpha-triggered application of TMS should demonstrate stronger ERS when applied to the DMN, as opposed to the DAN. Under the assumption that rest state favors DMN, we expect to observe longer relaxation times after DMN perturbation than the DAN perturbation. Conversely, if the relaxation times are not statistically different between TMS sites, the hypothesis that alpha power relates to DMN can be rejected. In the following, for both sites of stimulation, we report successively the TEPs and the induced alpha ERS/ERD.

## Results

### Pre-TMS Alpha baseline

The occipital alpha power is time and subject dependent, therefore the alpha power detection-threshold was defined per subject and allowed for slight changes during the experiment as described in the material and methods section. We compared the occipital alpha power (at the source level) for both stimulated sites (conditions) during the time period prior to the TMS (referred to as pre-TMS baseline; t = −0.4 to −0.1 s). We found that the occipital alpha power before TMS was not significantly different between both conditions (p > 0.05; mean ± standard deviation; MPFC: 1.45e-10 ± 4.53e-11; SPL: 1.52e-10 ± 5.27e-11).

### Alpha power during basic rest-state (basic αRest-State)

Resting state period was defined in the present data as the time window between t = −1.9 to −0.5 s prior to TMS for both conditions. The alpha power was averaged for each subject in this time window (mean ± standard deviation expressed as percentage of ERS/ERD; MPFC: −12.55% ± 8.39; SPL: −14.03% ± 7.47).

### TMS-Evoked potential (TEP)

**Figure 1:**
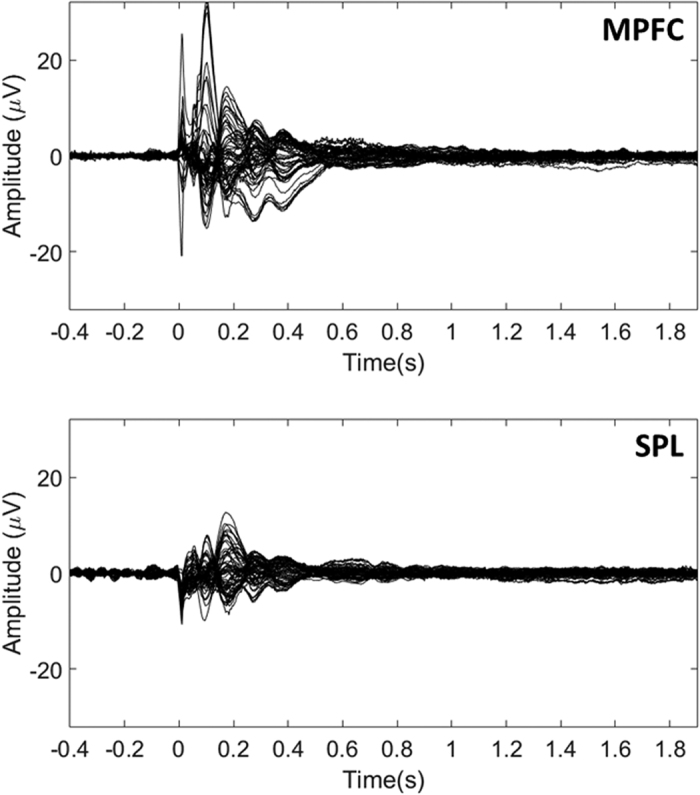
Grand average TMS-evoked potentials for both conditions (MPFC and SPL). Zero corresponds to the TMS onset in each condition.

Figure 1 presents the grand average TEP for both TMS conditions. TMS on both cortical sites evoked clear N100 and P200. Importantly, we note that evoked responses end at 0.5 s after TMS. The first “peak” around zero corresponds to the remains of the TMS-induced artifact, and was excluded from further analysis.

### Alpha Event-Related Synchronization/Desynchronization

**Figure 2:**
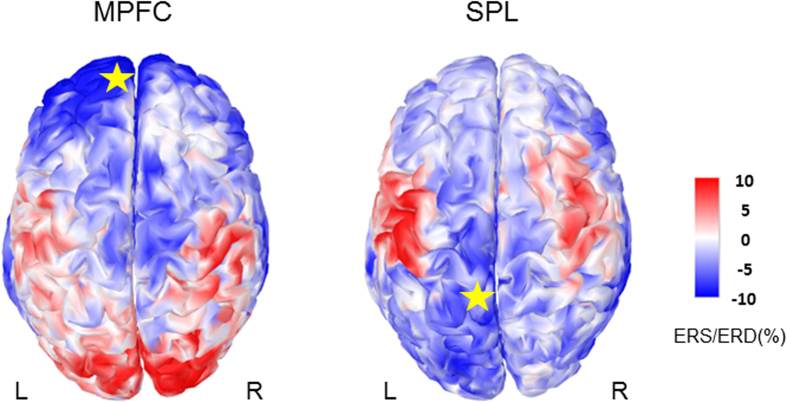
Grand average of the induced cortical alpha power after TMS on MPFC or SPL at t = 0.7 s with zero corresponding to the TMS onset. The alpha power is represented as a percentage of ERS/ERD relative to the pre-TMS baseline (t = −0.4 s to −0.1 s). The star indicates the stimulated site.

ERS and ERD were calculated with respect to the time period prior to the TMS (referred to as pre-TMS baseline, t = −0.4 to −0.1 s). Figure 2 presents the grand average of the cortical ERS/ERD following TMS over MPFC and SPL in the alpha band at t = 0.7 s after TMS, i.e. after the end of the TEP. There was clear ERS both in the occipital region and the centro-parietal regions. As we studied the induced alpha power in the occipital region, we defined an occipital region of interest (see Material and methods) and averaged the alpha power in this region, which we will refer to as αMPFC and αSPL for the two stimulated sites.

**Figure 3:**
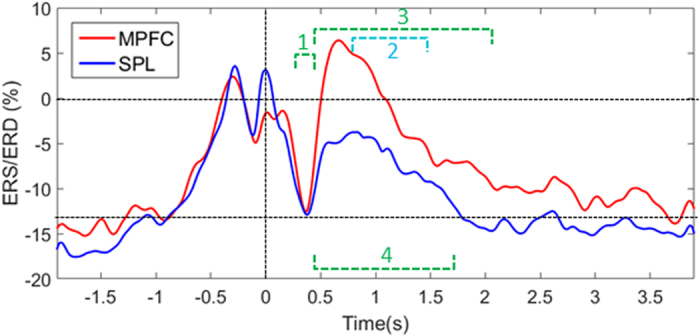
Grand average occipital alpha power (αMPFC, αSPL) across time around the TMS pulse (occurring at t = 0). The alpha power is represented as a percentage of ERS/ERD relative to the pre-TMS baseline (t = −0.4 s to −0.1 s). The mean basic alpha power during rest state period (αRest-state) for both conditions is represented as a dashed line. The annotated time periods mark: αSPL and αMPFC are not significantly different from αRest-state t = 0.28–0.45 s.αMPFC significantly higher than αSPL (p = 0.03) t = 0.78–1.44 s.αMPFC significantly higher than αRest-state (p = 0.0016) t = 0.48–2.07 s.αSPL significantly higher than αRest-state (p = 0.0207) t = 0.47–1.7 s.

On Fig. 3, the grand average of the time course of occipital αMPFC and αSPL is represented. The similarity of the alpha power during rest state period (t = −1.9 s to −0.5 s) for both conditions has to be noted (corresponding to the basic αRest-State). On Fig. 3, following TMS, there was a clear decrease of alpha power around t = 0.4 s, where the alpha power for both stimulated cortical sites is not significantly different from the basic αRest-State (p > 0.05, see segment 1 of Fig. 3).

Following this period, we notice an increase of alpha power, referred to as induced alpha power, which is significantly higher than basic αRest-state in both MPFC and SPL conditions (see segments 3 and 4 on Fig. 3; MPFC, p = 0.0016; SPL, p = 0.0207). Furthermore, we found that the occipital alpha power after TMS on MPFC (referred to as αMPFC) is significantly higher than the occipital alpha power after TMS on SPL (referred to as αSPL) (see segment 2 on Fig. 3, p = 0.03). Then, the alpha power decreases in both conditions and it returns sooner to the basic αRest-State for αSPL (around 1.7 s, see segment 4 on Fig. 3) than for αMPFC (around 2.1 s, see segment 3 on Fig. 3). We compared the duration of the induced alpha power in both conditions (see Materials and methods) and we found that αSPL lasts significantly shorter than αMPFC (p < 0.05; mean ± standard deviation of the duration in seconds, MPFC: 1.08 s ± 0.55; SPL: 0.8 s ± 0.48)

## Discussion

In the present study, we used single pulse TMS to test the hypothesis that alpha power is linked to the DMN; we transiently perturb brain dynamics at one node of the DMN or one node of the DAN and investigated the transient brain dynamics relaxing towards the resting state. TMS targeted either MPFC, a node of the DMN, or SPL, often cited as a part of the DAN^5^,^6^. TMS was triggered by high occipital alpha activity, under the hypothesis that the DMN was preferentially activated^26^. We analysed the brain’s response to TMS in terms of TEP, as an index of system’s perturbation, and induced alpha ERD/ERS. We observed four main results: 1) TEPs were of greater magnitude for MPFC than for SPL, 2) in both conditions, throughout the period of TEP, an initial decrease in occipital alpha power returned the system to the basic alpha rest-state level, 3) after this return to the basic alpha level, a long lasting increase in occipital alpha power was observed and 4) this occipital alpha ERS was significantly higher and longer lasting when stimulating MPFC (node of the DMN) than when stimulating SPL (node of the DAN), this reinforces the hypothesis of a strong coupling between DMN and occipital alpha power.

Although the stimulation intensity was similar, the observed TEP was of greater magnitude for MPFC than for SPL. However, the pre-frontal cortex is generally known to be less excitable than, at least, the motor cortex^38^. This shows that MPFC excitability is higher than SPL excitability when the occipital alpha is high and argues in favor of a link between MPFC and the alpha generation process. In both cases, this initial TEP corresponded to a decrease in occipital alpha power with respect to the pre-TMS baseline. The αMPFC and the αSPL return to the basic alpha power level (referred as αRest-state, see Fig. 3). Because this moment coincides with the end of the ERP, it was considered as a reset of the alpha power^19^.

After this reset, the induced αMPFC and αSPL behave like an Event Related Synchronization (ERS) with respect to the basic αRest-state and with a higher occipital ERS for MPFC (see segment 2 Fig. 3). Moreover, the induced αMPFC lasted more than 2 s (see segment 3 on Fig. 3) while, for SPL, TMS induced an alpha ERS smaller and also with a shorter duration. Only two studies characterized the oscillatory response to single pulse TMS over M1 during rest^39^,^40^. They reported a short-lasting alpha ERS in the first 500 ms following TMS but in the following 500 ms the oscillatory response disappeared, even if the stimulation intensity is increased until 130% of the resting motor threshold^39^. Similarly, Paus and collaborators^40^ reported a brief period of synchronized activity in the beta range. On the other hand, several studies emphasized that alpha generation is a typical response to repetitive TMS; among other 1 Hz-rTMS^21^ or 20 Hz-rTMS^41^ over M1 that most of the time is restricted to the stimulated zone. More recently, rhythmic TMS (5 pulses at alpha rhythm) has been found to cause local entrainment of the alpha rhythm, mainly during the first two pulses of the 5 pulses train^42^, i.e. over a few hundred ms. Novel findings demonstrate, for the first time, alpha induced responses lasting up to 1 seconds following single pulse TMS, despite modest stimulation intensity. This prolonged effect is found not only in the vicinity of the stimulated sites but also in distal ipsilateral regions as well as contralateral regions (see Fig. 2) as if we had interfered with the alpha generation process. To explain the bilaterality of the occipital ERD/ERS, we note that TMS was applied close to the medial line, at the level of the interhemispheric sulcus, thus most likely both sides were affected by stimulation even if the stimulation was gentle. Nevertheless, the effect may also reflect the bilaterality of the DMN^1^,^2^ and the strong interhemispheric connectivity. Since we stimulated the left hemisphere in right handed persons, this could still reinforce the phenomenon.

Previous studies showing a link between DMN and the posterior alpha activity observed during the resting state, relied on correlative techniques, showing links between EEG^2^ or MEG activity^3^,^16^ and DMN activation. Recent studies have also associated the DMN specifically with the alpha band^24^,^26^. With combined EEG/fMRI, it was shown that posterior alpha was positively correlated with BOLD activity in the DMN during the resting state^24^,^26^. However, a correlation does not establish any causal link^35^,^36^ between alpha power and DMN. The present study sought to establish such a mechanistic association: by applying a transient stimulation to the MPFC, i.e. a node of the DMN, we induced a prolonged alpha ERS as if the stimulation of one node of the DMN during its “activation” had boosted the occipital alpha power, which can be interpreted in terms of reinforcement of the DMN activation. This further suggests that MPFC, and possibly the DMN, plays a mechanistic role in the alpha generation process.

DMN and DAN respectively participate in the “task-negative” and the “task-positive” networks. These two diametrically opposed, widely distributed brain networks show both spontaneous correlations within each networks and anticorrelations between networks^12^. This perspective emphasizes the role of a brain region in its function as a network node, in which the global network character supersedes the local region character for brain dynamics. In the present study, we observed ERS in responses to single pulse TMS in both cases, while stimulating an area from the DMN and an area from the DAN with similar stimulation intensities. We note that SPL corresponds to the region that Thut and collaborators found as an active generator of alpha oscillation during an activation paradigm of spatial attention^42^; this could explain why we also observed an alpha ERS following single pulse TMS over this site. However, the results show that the induced alpha power following TMS on a node of the DAN, when the DMN is dominant in the resting state at the time of the stimulation, is significantly smaller and takes less time to return to the αRest-state level than the induced alpha power following TMS on a node of the DMN. The difference in this slow, long lasting effect is clear evidence of a stronger coupling between alpha rhythm and DMN compared to DAN, which strongly suggests that MPFC is indeed involved in the alpha generation process during resting state.

The brain resting states are routinely investigated in clinical research, moreover they are very frequent in daily life; however, their underlying dynamics is largely unknown. We have here provided further support that MPFC is more embedded in the network producing the alpha rhythm than SPL. Concerning the implication of this finding, nowadays, rhythmic TMS protocols are more and more used in order to modulate brain function with the idea that they allow to improve the TMS effects in comparison with standard rTMS protocols^42^,^43^,^44^. Following TBS protocols, alpha burst protocols are currently in development, and indeed they have been shown to modulate cognitive functions such as visual perception^45^. Moreover, several authors proposed to trigger the stimulation trains depending on background EEG activity (the alpha level in particular) to further improve their effects^45^,^46^,^47^. The present experiment showed that even single pulse TMS is able to induce long lasting occipital alpha rhythm modulations if triggered by occipital alpha; this could reflect the basic mechanism underlying the efficiency of rhythmic alpha TMS protocols^42^,^44^. Our results provide confirmation of the existence of long-lasting transient patterns after single pulse stimulation, and highlights the unique role of DMN in the organization of the resting state.

## Methods

### Subjects

Ten right-handed subjects (4 men and 6 women), from 22 to 30 years old, participated in this experiment. All subjects gave their full and written informed consent, after having been told about the experimental procedure and TMS. None had neurological antecedents or any contraindication to TMS. This study was approved by the local ethical committee (CPP Sud-Méditerranée I) and was in accordance with the declaration of Helsinki.

### Stereotaxic TMS

**Figure 4:**
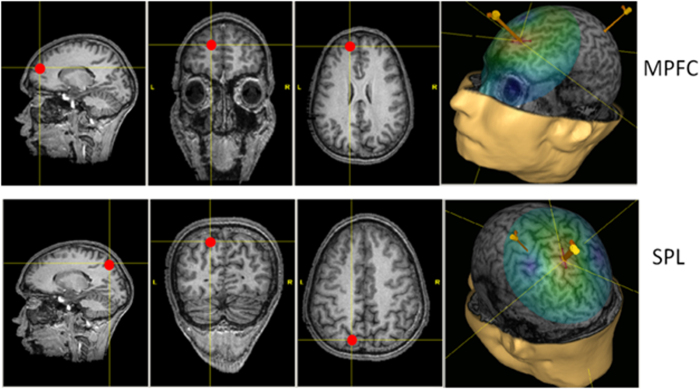
For each stimulation condition, the targeted point is shown on MRI slices by the red dot. An example of the induced electric field represented on a 3D reconstruction of the brain is also shown for each stimulated site, with the hand area in the primary motor cortex as a reference point. For left MPFC, we targeted the area BA10, i.e. anterior part of the superior frontal gyrus, very near the median line. The coil handle pointed backward with an angle of approximately 10° with respect to the midline. For left SPL, we targeted area BA7, i.e. part of the parietal cortex, located between the marginal branch of the cingulate sulcus and the parieto-occipital sulcus. The coil handle pointed towards the back of the head, forming a 45° angle with respect to the midline in the clockwise direction.

We used a Magstim 200 stimulator (Magstim Company, Whitland, UK) that generates a monophasic magnetic field of up to 1.7 Tesla, connected to a coplanar figure-eight coil with an external loop diameter of 9 cm. The coil was maintained in the desired position by a custom apparatus. The coil could thus be moved or placed optimally, while keeping its position stable throughout the experiment. The stimulation system was connected to a neuro-navigation device (Navigation Brain System 2.3, Nexstim, Helsinki, Finland), using anatomical magnetic resonance imaging (MRI) scans (T1) of each subject to precisely guide the stimulation. The system computes an estimate of the electric field induced in the cortex by the TMS pulse in real time (depending on the site’s depth among others) and displays it on the subject’s anatomical MRI. Using this system, the coil was placed to stimulate one of two cortical areas: the left medial prefrontal cortex (MPFC) or the left superior parietal lobule (SPL) in two successive sessions. These sites were defined based on individual anatomical landmarks (see the legend of Fig. 4), and then translated into the MNI coordinates system. For left MPFC, mean (±std) MNI coordinates (x, y, z) of the stimulated point were (−9 ± 8, 55 ± 3, 17 ± 5). For left SPL, mean (±std) MNI coordinates (x, y, z) of the stimulated point were (−8 ± 3, −67 ± 6, 53 ± 4).

For the first site, the stimulator intensity was initially tuned to induce a distinguishable TEP in the surrounding electrodes, meaning that a minimum threshold of 7 μV had to be met for the N100 component in the first 5 trials. Then, for the second site, the stimulation intensity was set at the same level as the stimulation intensity for the first site because the depths of the chosen targets (observed on MRI) were similar^48^. For MPFC, the mean stimulation intensity was 59.6 ± 6.0% (mean ± standard deviation) of the maximum stimulator output and 58.7 ± 8% for SPL (t(1,9) = 0.35, P > 0.05).

### Experimental set-up and protocol

Subjects sat on a semi-reclined chair, with forearms lying on armrests. Eyes were closed, and covered with cotton pieces to isolate the subject from light and avoid blinks. Care was taken so that subjects could stay relaxed. To limit the subjects from hearing the ambient noise and the sound made by the coil discharge, they wore ear plugs. They were instructed to stay motionless without falling asleep and “to let their thoughts move freely without engaging in any particular task”. Throughout the experiment, we continuously verified that the subjects were still awake by online inspection of the EEG data, and if not we touched his/her shoulder.

The experiment consisted of two experimental TMS sessions corresponding to the two targeted areas, 1) MPFC and 2) SPL. The order of the TMS sessions was counter-balanced; five subjects began with SPL stimulation and five with MPFC stimulation. Before starting the experiment, 4 minutes of rest without TMS were recorded, allowing us to observe the initial resting state dynamics (without TMS) and to select the electrode near the occipital region (often POz) where clear alpha activity was observed. The first alpha detection-threshold to be used was quantified on this electrode and used to define the initial alpha range of each individual (see supplementary information). Then the first TMS session began, during which the EEG from the selected electrode was analysed on-line (using the BrainVision Recview interface, BrainProducts, Gilching, Germany). Alpha bursts were detected with a Fast Fourier Transform (FFT) based algorithm (non-overlapping windows of 250 ms), and TMS was triggered automatically when the power in the 8–13 Hz band exceeded the initially-determined threshold. Thus, TMS was applied at high occipital alpha power for each subject. Following a TMS pulse, a minimum inter-stimulus of five seconds ensued, under the hypothesis that the stimulus induced transient dies out and the brain returns to rest state dynamics within this interval. If needed, the alpha detection-threshold was slightly changed during the experiment to adapt to the ongoing alpha level of each subject. For each stimulated site, two blocks were run during which 45 TMS pulses were applied.

### Data recording

The EEG was recorded continuously with TMS compatible equipment (BrainAMP-DirectCurrent, BrainProducts, Gilching, Germany) with a sampling frequency of 5 kHz, using BrainVision Recorder software. We used a 62-electrode cap (Fast&Easy EasyCap) with Ag/AgCl electrode material, mounted on an elastic cap positioned according to the 10–20 method extended to 64 electrodes. In the vicinity of the coil, the cable of each electrode was tilted so that it is approximately perpendicular to the coil cable to limit the TMS-induced artefact on the EEG signal. The ground electrode was positioned on the forehead and the reference was placed on the right mastoid. Skin-electrode impedance was maintained below 10 kΩ. A one-centimeter plastic foam was attached below the coil to prevent it from touching the electrodes. The characteristics of the electric field induced by each TMS pulse (localization, orientation and intensity) were recorded by the neuro-navigation device.

### Data analysis

#### Preprocessing

EEG data were first average referenced and downsampled to 2,5 kHz using the BrainVision Analyzer 2.0 software. Data were segmented in trials of 6 seconds (2 s before, 4 s after TMS). The trials were then exported to MATLAB (The Mathworks, Inc.) in the EEGLAB 12 toolbox^49^ for eye inspection. Trials with artifacts (originating from muscle activity, movements or electrical noise) were rejected; 72 ± 8 trials were kept for the condition SPL and 68 ± 9 trials for the condition MPFC (with 41 as the minimum). The stimulation artefact was removed in each trial using a custom script cutting the signal from −5 to 10 ms and replacing it by a straight line with added white noise (see supplementary information). The signal was then filtered using a 0.5 Hz High-Pass Butterworth filter (order 2) to correct eventual signal drifts.

The anatomical MRI (T1) for each subject was treated with the FreeSurfer software^50^ to obtain automatic volumetric segmentation and cortical parcellation. The results were visually inspected to check that no defects were present.

Functional and anatomical data were then imported in Brainstorm, a freely available software^51^, for source analysis. To save memory, EEG data were down-sampled to 512 Hz. A notch filter was only applied to EEG data for the TMS-Evoked Potentials (TEPs) visualization.

### Spectral analysis

As EEG offers a limited spatial resolution, we chose to work at the source level instead of electrode level. To estimate the spatiotemporal activity of the neural sources of the EEG data, we first computed the forward modelling, using the OpenMEEG Boundary Element Model^52^. For the inverse modelling, we used the whitened and depth-weighted linear L2-minimum norm estimates implemented in Brainstorm^51^. The estimated sources orientations were constrained to be perpendicular to the cortical surface.

As we focused on the Event-Related Synchronizations (ERS) or desynchronizations (ERD) in the alpha 8–13 Hz band^35^, alpha power was obtained from source estimates by taking the absolute value of the Hilbert transform of the source estimate bandpass filtered in the alpha band (8–13 Hz). Power over all trials was averaged for each condition and each subject, and normalized with respect to the time period prior to the TMS (referred to as pre-TMS baseline; t = −0.4 to −0.1 s). Here, power increases or decreases are reported with respect to this baseline as ERS or ERD respectively. It should be noted that this baseline corresponds to periods of high alpha activity with the idea to favour the involvement of the DMN, therefore it is higher than the basic alpha activity during rest state period (referred to as αRest-state; t = −1.9 s to 0.5 s before TMS). For averaging the cortical activations across subjects, we projected the ERS/ERD from each individual anatomy onto the Colin 27 average brain^53^. To study the occipital alpha ERS/ERD, an occipital scout was defined based on the Destrieux atlas^54^ and composed of the sum of the occipital pole, middle and superior occipital gyrus, middle occipital sulcus and lunatus sulcus, superior occipital sulcus and transverse occipital sulcus for each hemisphere. This aggregate occipital scout has a surface area of 155 cm^2^ in the Colin 27 brain.

### Statistical Analysis

For each subject, we compared the occipital alpha power before TMS averaged across trials between both stimulated cortical areas, left MPFC and left SPL. For this purpose, we used the Mann-Whitney U test implemented in MATLAB (The Mathworks, Inc.). Then, to compare the occipital alpha ERS/ERD between both stimulated cortical areas, we extracted the average time courses of the cortical activations in the defined occipital scout for each subject and performed the statistical inference on this average at the subject group level. Using a non-parametric permutation test based on clustering in the temporal domain implemented in the Fieldtrip toolbox^55^, we compared the occipital alpha power after TMS between stimulation sites. Moreover, we tested whether the alpha power after TMS on both sites were significantly different from basic αRest-state (alpha power during rest-state, t = −1.9 s to −0.5 s). For each comparison, the Monte Carlo approximation used 10000 permutations. The significance level was set to a two sided p-value of 0.05 for the clustering. A t-test for dependent samples was used as first-level statistic. This test allows a correction of the Multiple Comparison Problem while accounting for the temporal adjacency in the data^56^. Finally, to compare the duration of the induced alpha power following the TMS, the alpha power for each individual and each stimulated cortical area was first standardised with respect to each basic αRest-state then compared to the 5% threshold (1.96 * σ). The time period for which the z-scored alpha power is higher than this threshold corresponds to the duration of the induced alpha power. This duration was determined for each subject and each condition and we used a t-test to compare those durations between conditions.

## Additional Information

**How to cite this article**: Bonnard, M. *et al*. Resting state brain dynamics and its transients: a combined TMS-EEG study. *Sci. Rep.* **6**, 31220; doi: 10.1038/srep31220 (2016).

## Supplementary Material

Supplementary Information
