# Supplementary material for: Resting state brain dynamics and its transients: a combined TMS-EEG study
Source: Sci Rep. 2016 Aug 4;6:31220. doi: 10.1038/srep31220 (PMC4973226; doi:10.1038/srep31220)
Supplement: Supplementary Information [file srep31220-s1.pdf]

Additional information

Resting state brain dynamics and its transients: a combined TMS-EEG study

Mireille BONNARD*, Sophie CHEN, Jérôme GAYCHET, Marcel CARRERE, Marmaduke WOODMAN, Bernard GIUSIANO & Viktor JIRSA

Online alpha-detection procedure triggering TMS

During the experiment, the alpha rhythm was analyzed online on POz based on FFT run over a 250ms time window using the Recview interface (BrainProduct, Gilching, Germany) as shown in the figure below. Whenever the power of the alpha exceeds the individually-defined alpha detection threshold, TMS was triggered using the 'Band-Power-Trigger' procedure that send signals to the PC's parallel port (connected to the TMS device). According to the subjects, this threshold corresponds to 10-15% of variation with respect to the basic alpha rest-state (10%<ERS<15%, see figure 3).

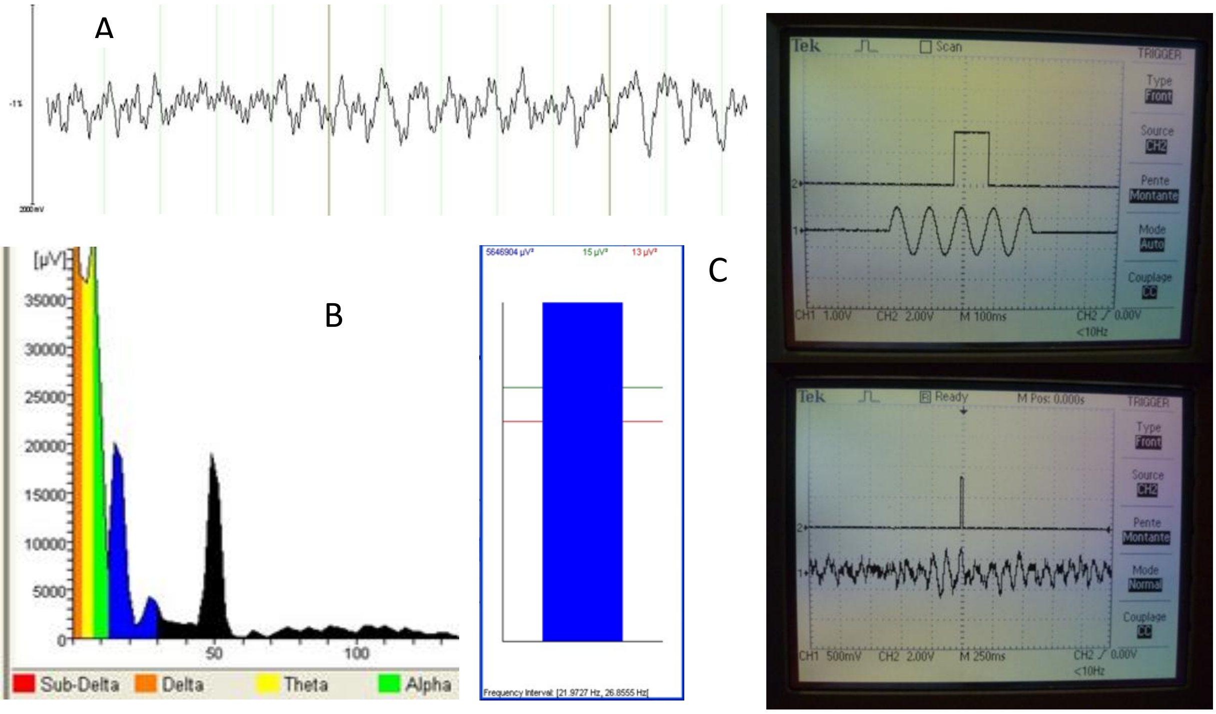

Main stages of the online alpha-detection procedure. A. Raw EEG signal on POz electrodes. B. FFT plot for the different frequency bands. C. Online visualization of the alpha power, horizontal lines (min and max of the threshold value) were controlled with sliders. Figures on the right panel shows the TTL pulse triggered in case of a test signal (top panel) or a real EEG signal (bottom panel).

The Band-Power-Trigger menu is equipped with two slide control (see figure C) that enable the experimenter to adjust the setting of the alpha detection threshold graphically at any time during monitoring or recording modes. Time delay after alpha detection is evaluated around 40ms. After triggering a TMS pulse, a pause of 5 sec allowed the system to return to the basic alpha rest state.

Effect of artifact removal

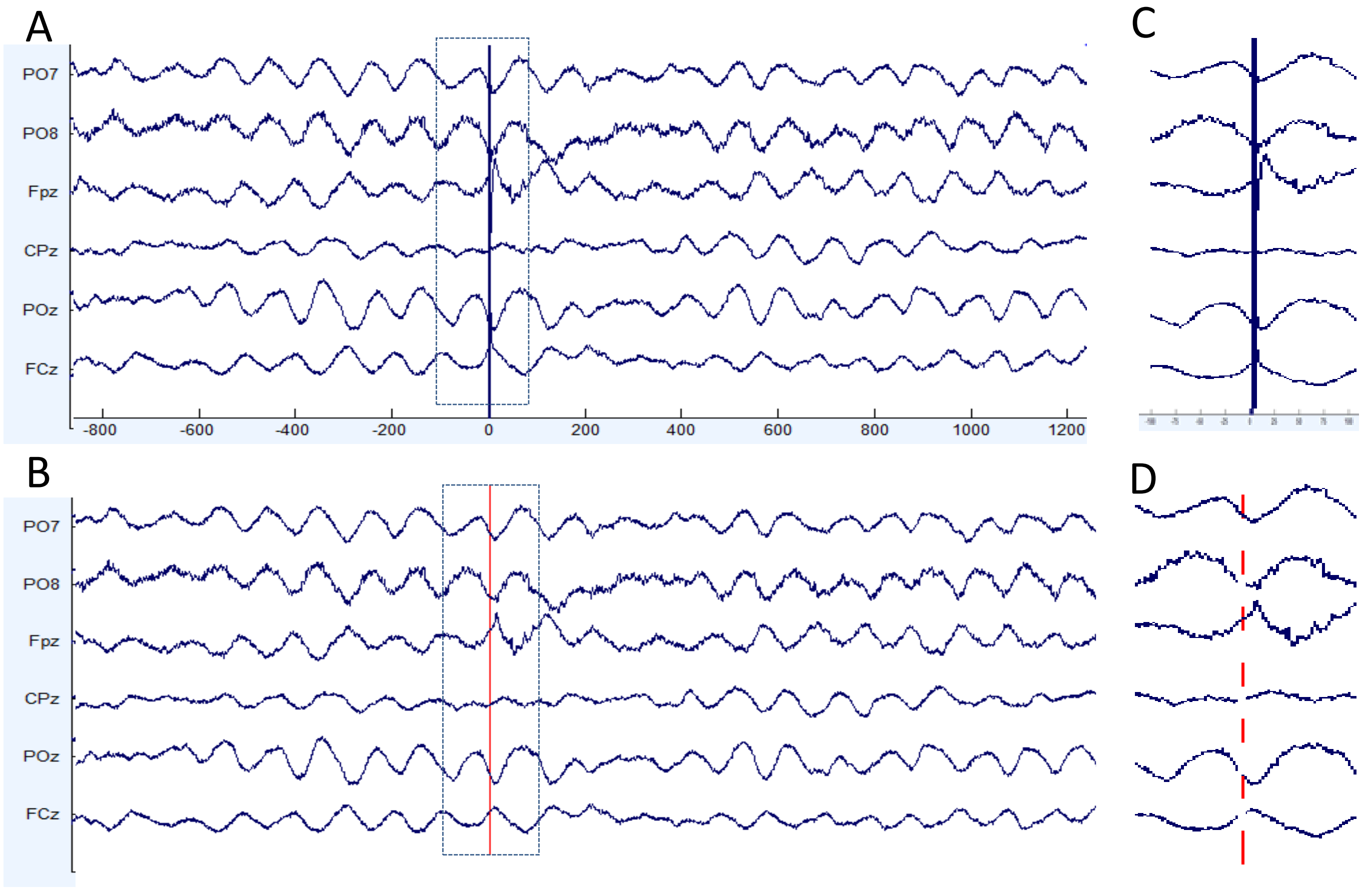

Raw EEG signal during a typical trial (A) on 6 electrodes from 0.1 s before TMS to 1.2 s after TMS. The same data after artifact removal (B). C and D are an enlargement of the data from -150 ms to 150 ms around TMS: raw data (C) and after artifact removal (D).
